# Surviving the Tsunami of Receiving a ‘Goodbye’ Note

**DOI:** 10.1111/jpm.70120

**Published:** 2026-03-18

**Authors:** Roya Haghiri‐Vijeh, Archana Paul

**Affiliations:** ^1^ School of Nursing, Faculty of Health York University Toronto Ontario Canada

## 1 Introduction

The individual who attempted suicide often leaves a suicide letter for family members before they take their own life (Hennipman‐Herweijer et al. 2024; Lang et al. 2021; Furqan et al. 2019). The suicide letter, also known as a *goodbye* or a *suicide note*, may include the reasons behind the individual's decision to attempt suicide, seek forgiveness, express contentment with the decision, blame others for their experiences and/or provide instructions on how to inform others about their last rites (Gunn et al. 2025). In some cases, the individual may ask the recipient of the letter to conceal the reason of death being suicide (Vivekanandhan et al. 2024; Eilers and Kasten 2022; Furqan et al. 2019). The suicide letter may be addressed to a specific individual or conveyed more generally. Upon discovering a suicide letter from their loved ones, family members often enter a survival mode, where they make every effort to assist and support the individual who attempted suicide (Vatne et al. 2021).

This narrative article will begin with the first‐person lived experience of receiving a suicide letter, written to the first author by the individual who attempted suicide. Thereafter, drawing on our experiences in mental health nursing practices, we will share our current understanding of the gaps in practice and provide key takeaway messages for approaches to support family members who have received the suicide letter. In this article, we are not claiming that the recipients of a suicide letter are more or less affected compared to family members who were not recipients of the letter; rather, we are shedding light that recipients of the letter may need unique follow‐up and support from social and healthcare professionals. Before we present our experience, insights, and recommendations for social and healthcare providers, we begin by clarifying the terms family and the individual who attempted suicide. The term *family* is used as an umbrella term to encompass an individual born into a family, chosen family and friends who have a close relationship with this individual who attempted suicide. We are using the phrase *individual who attempted suicide* to pay attention to the first‐person experiences and avoid blaming language.

A holistic lens guides this narrative piece, where it situates experiences as interconnected and impacting each person involved in a phenomenon. Holistic nursing care acknowledges a patient‐centred care approach and highlights that experiences are not distinct, and care providers need to account for the physical, social, spiritual and psychological needs of the patient (DeBastiani and De Santis 2017). Family members are a source of social support for patients, who often continue care after they are discharged from the healthcare facility (Hennipman‐Herweijer et al. 2024; Vatne et al. 2021). As such, the mental health of the recipients of the suicide letter—whether a family member or close friend—needs to be included as part of the treatment and recovery plan. A gap remains in clinical practice concerning support for the recipient of a suicide letter, as well as for the families of individuals who have attempted suicide. The challenges faced by the recipients are encountered globally and have a significantly negative impact on their well‐being (Vivekanandhan et al. 2024; Hennipman‐Herweijer et al. 2024). We have offered the key takeaway messages for social and healthcare providers as they engage with the recipient and the family members of individuals who have attempted suicide.

## 2 Experience of Receiving the Suicide Letter

Although a physical suicide letter is often left for the family, I, the first author, had a different experience of receiving the letter. I received the *suicide* note through an email communication while on a vacation outside of my home country. Let's provide some context about my positionality and role. After enduring several months of stressful career transitions and family obligations, I was on a brief two‐day respite for an overseas holiday with my children, but without my spouse. As I happened to check my phone, I received an email from this individual with whom I had not been in contact for a few months. Due to conflicting schedules, this individual would not normally contact me regularly. The subject line of the email read, ‘With Great Gratitude’. What does this mean? I thought to myself and opened the email with curiosity and some nervousness. What followed immediately was a tsunami of shock, confusion, guilt and helplessness. Adding to this unsettling situation was the fact that I was in a different country. After a few seconds of shock, which felt like a lifetime, I contacted another family member in my home country and promptly directed them to call 911. Within a few minutes, I was required to assume control of the situation and operate as both a director and a navigator, endeavouring to soothe the anxious family members, coordinating with the police, paramedics and healthcare professionals, all while attending to my young family members. After a couple of days, I was able to return to my home country and coordinate further meetings with the social and healthcare providers who provided care for this individual who attempted suicide, while attending to my job deliverables and taking care of a sick child.

In this real‐life event, the individual who attempted suicide was transferred to the intensive care unit from the emergency room, where they were monitored for 4 days. While at the hospital, at home or at work, I was consistently interacting with social and healthcare providers. However, there was no clear concern shown by these providers about my well‐being as I continued to experience feelings of guilt, shame and helplessness after the event. Once stable, the individual who attempted suicide was transferred to the medicine floor and referred to the mental health consultation team to receive further support. The Psychiatrist did not assess this individual for the next 4 days. I, along with other family members, had no idea about the treatment plan and continued to wait patiently to hear about the next steps from the nurses on the floor. Similar to previous studies, I found this lack of coordination between teams and services added to the unnecessary burden and led to a lack of holistic care in the psychiatric and mental health unit (Marshall et al. 2023; Vatne et al. 2021). From my experience, I found this to be an obstacle in the continuity of care and further amplified my uncertainty (Marek and Oexle 2025).

## 3 Why Are We Sharing This Experience?

It is crucial to situate the positionality of the recipient. The recipient has multiple intersecting experiences as a past refugee, newcomer, first‐generation settler, gender‐minority and one who has experienced trauma and violence in the past. Throughout their interactions with various members of the social and healthcare provider team, the conversation predominantly focused on the immediate needs of this individual who attempted suicide. This is a natural course of action (Vatne et al. 2021); however, *not even once* did any members of the social and healthcare provider team ask the recipient a simple question, ‘How are you doing?’ or ‘Receiving the letter must have been difficult. Do you have resources or support that are easily accessible?’ As a healthcare professional and due to their previous knowledge and experience, the recipient was aware of their mental health status and was well informed of paid and free counselling services to address their own mental health and psychological needs. Yet, to reach this point of realising the need for counselling support, it took a few weeks of emotional turmoil for the recipient, which manifested as physical pains and aches, which in turn impacted their productivity personally and professionally. Spillane et al. (2018) indicate that family members affected by the suicide attempt of their loved one experience deteriorated physical and mental health, including increased rates of outpatient physician visits.

## 4 Call to Action for Psychiatric and Mental Health Nursing

The suicide letter may entail suicidal ideations, plans, intent and access to means in attempting suicide (Furqan et al. 2019). Attention is primarily focused on this individual, often leaving the needs of the recipient unaddressed. This gap in practice is concerning, as family members, including the recipient, require support to cope with the aftermath of such an event (Vatne et al. 2021). Based on our current experience in practice, some ongoing gaps include social and healthcare providers not recognising the impact of being the recipient of the suicide letter, the recipient's coping with this devastating situation, and their lack of involvement in discharge planning. This adds an unnecessary burden as they (family members and the recipient) are left to navigate the situation on their own. The constant fear of the possibility of encountering a similar situation, maintaining a high sense of vigilance, and the impact on their own well‐being, in addition to other roles and responsibilities, can be extremely stressful (Marshall et al. 2023; Vatne et al. 2021, 2023).

## 5 Key Takeaway Messages for Psychiatric and Mental Health Care Providers

1. Listening to the unheard voices: The unheard voices of the family members and the recipient need to be amplified to bring changes in the current practice. Social and healthcare providers must be trained to recognise, acknowledge, meaningfully ask and listen to the family members about the impact of suicide in a respectful and non‐judgmental manner (Marshall et al. 2023; Vatne et al. 2021, 2023). When possible, family members must be engaged in the discharge planning, and their recommendations must be the centre of the care to foster a culture of shared decision‐making.
2. Coordinating between healthcare teams and services: A collaborative approach between all social and healthcare providers will alleviate the additional stress on the family members and the recipient. Training on addressing the needs of the family members must be mandatory. This training should focus on strengthening core competencies, including cultivating empathetic attitudes and self‐awareness toward family members, conducting thorough assessments, implementing effective interventions and developing safety plans as necessary (Marek and Oexle 2025; Registered Nurses Association of Ontario 2009). Additionally, the training should aim to foster hope and promote a sense of recovery among both patients and their families (Vatne et al. 2021). In a team approach, a designated social and healthcare provider, such as a mental health nurse, should be assigned to follow up and update the recipient on this individual's day‐to‐day progress. Prompt and transparent communication regarding any emergent medical condition resulting from the suicide attempt, as well as associated consultations and referrals, is imperative to keep the recipient and the family updated (National Institute for Health and Care Excellence 2022).
3. Availability of sufficient resources: Social and healthcare providers must have access to readily available resources to share with the recipient and family members, outlining the next steps, community supports and emotional well‐being (Marshall et al. 2023). This resource must guide to answer questions such as, ‘what should I do if I encounter a similar situation in future’, ‘what about my mental health’ and ‘what can I learn from others who have been in a similar situation’.
4. Following up: It is crucial to follow up with the recipient of the letter regularly via phone call or email to inquire about their well‐being. A checklist of items to follow up could be attached to this individual's (who attempted suicide) electronic and/or paper chart and must focus on providing basic education on the recipient's mental health needs, understanding and moving through trauma, guilt and grief, healthy coping strategies, accessing community resources, including a 24/7 telehealth and counselling support.
5. Suicide prevention task force: Hospitals must establish a suicide prevention task force that includes nurses, social workers, physicians, peer support workers, and crisis and addictions workers. This task force shall be responsible for follow‐up and the provision of ongoing counselling to individuals who have attempted suicide, as well as to their family members, including the recipient of the suicide letter. It is recommended that social and healthcare providers with expertise in mental health and crisis prevention oversee the activities of this task force (Marek and Oexle 2025; Perlman et al. 2011).

## 6 Conclusion

This narrative article delves into the real‐life experiences of the recipient of a g*oodbye* note who continues to support the individual who attempted suicide. In addition to the health and safety of the individuals who attempted suicide, social and healthcare providers need to assess and support the well‐being of the recipient of the suicide letter. Ongoing research and sustained engagement with the recipient and this individual, including the social and healthcare providers, are essential to develop, pilot and evaluate interventions aimed to support them.

## Conflicts of Interest

The authors declare no conflicts of interest.

## Data Availability

The authors have nothing to report.
